# Effectiveness of Nifurtimox Eflornithine Combination Therapy (NECT) in *T*. *b*. *gambiense* second stage sleeping sickness patients in the Democratic Republic of Congo: Report from a field study

**DOI:** 10.1371/journal.pntd.0009903

**Published:** 2021-11-08

**Authors:** Andrea Kuemmerle, Caecilia Schmid, Sonja Bernhard, Victor Kande, Wilfried Mutombo, Medard Ilunga, Ismael Lumpungu, Sylvain Mutanda, Pathou Nganzobo, Digas Ngolo Tete, Mays Kisala, Christian Burri, Severine Blesson, Olaf Valverde Mordt

**Affiliations:** 1 Swiss Tropical and Public Health Institute, Basel, Switzerland; 2 University of Basel, Basel, Switzerland; 3 Programme National de Lutte contre la Trypanosomiase Humaine Africaine (PNLTHA), Kinshasa, Democratic Republic of the Congo; 4 Drugs for Neglected Diseases *initiative*, Geneva, Switzerland; 5 Bureau Diocesain d’Oeuvres Médicales (BDOM), Kikwit, Democratic Republic of the Congo; Institute of Tropical Medicine, BELGIUM

## Abstract

**Background:**

Nifurtimox-eflornithine combination therapy (NECT) for the treatment of second stage gambiense human African trypanosomiasis (HAT) was added to the World Health Organization’s Essential Medicines List in 2009 after demonstration of its non-inferior efficacy compared to eflornithine therapy. A study of NECT use in the field showed acceptable safety and high efficacy until hospital discharge in a wide population, including children, pregnant and breastfeeding women, and patients with a HAT treatment history. We present here the effectiveness results after the 24-month follow-up visit.

**Methodology/Principal findings:**

In a multicenter, open label, single arm phase IIIb study, second stage gambiense HAT patients were treated with NECT in the Democratic Republic of Congo. Clinical cure was defined 24 months after treatment as survival without clinical and/or parasitological signs of HAT. Of the 629 included patients, 619 (98.4%) were discharged alive after treatment and were examined for the presence of trypanosomes, white blood cell count in cerebro-spinal fluid, and disease symptoms. The clinical cure rate of 94.1% was comparable for all subpopulations analyzed at the 24-month follow-up visit. Self-reported adverse events during follow-up were few and concerned mainly nervous system disorders, infections, and gastro-intestinal disorders. Overall, 28 patients (4.3%) died during the course of the trial. The death of 16 of the 18 patients who died during the follow-up period was assessed as unlikely or not related to NECT. Within 24 months, eight patients (1.3%) relapsed and received rescue treatment. Sixteen patients were completely lost to follow-up.

**Conclusions/Significance:**

NECT treatment administered under field conditions was effective and sufficiently well tolerated, no major concern arose for children or pregnant or breastfeeding women. Patients with a previous HAT treatment history had the same response as those who were naïve. In conclusion, NECT was confirmed as effective and appropriate for use in a broad population, including vulnerable subpopulations.

**Trial registration:**

The trial is registered at ClinicalTrials.gov, number NCT00906880.

## Author summary

The advanced stage of the neglected tropical disease human African trypanosomiasis was, until relatively recently, treated with an old toxic arsenical drug and there was little investment in an improved treatment option. Eflornithine alone was efficacious, but difficult to administer as it required four two-hour infusions a day for 14 days. Nifurtimox-eflornithine combination therapy (NECT) was developed as a simplified and easier to use treatment and was shown to be effective and sufficiently well tolerated in a randomized clinical trial. The present study was conducted to assess the overall effectiveness, including the feasibility of implementation of NECT under field conditions in a wider population than in the randomized clinical trial. We found that NECT can be implemented under field conditions and in remote areas, with the necessary logistical support and staff training for treatment administration. Adverse events, although very frequent, were considered acceptable given the severity of the disease. Less than 10% of patients showed severe adverse events. Over 24 months, the case fatality rate was 4.5% and relapses were rare (1.3%). The effectiveness of NECT was confirmed in a broad spectrum of second stage gambiense HAT patients, including children, pregnant and breastfeeding women, and patients who had been previously treated for HAT.

## Introduction

Human African trypanosomiasis (HAT) is a neglected tropical disease (NTD) caused by the protozoan parasite *Trypanosoma brucei* transmitted by the tsetse fly. The West African form, *T*. *b*. *gambiense* accounts for the majority of currently reported HAT cases. This disease, which is fatal in almost all cases if untreated, progresses from a first haemolymphatic stage to a second meningoencephalitic stage, which ultimately leads to severe sleep disturbances, other neurological and psychiatric disorders, coma, and eventually death [1,2].

HAT affects people living in sub-Saharan Africa, with most cases diagnosed and reported in the Democratic Republic of Congo (DRC), mostly in remote and/or insecure areas with limited access to health care [3]. For decades, HAT control suffered from a lack of acceptable control tools and insufficient research efforts, resulting in a transmission peak reached in the late 1990s [4]. Since then, constant surveillance and control activities have brought the epidemic under control with less than 1’000 HAT patients reported annually since 2018 [5].

Until 2009, treatment for the second stage of *T*. *b*. *gambiense* HAT was limited to melarsoprol, a toxic arsenic derivative, or eflornithine, with a burdensome treatment administration requiring 56 slow infusions administered over 14 consecutive days [6]. A new treatment alternative, nifurtimox-eflornithine combination therapy (NECT) consisting of the administration of only 14 slow infusions administered every 12 hours for 1 week, and a concurrent 10-day oral treatment with nifurtimox, was developed and subsequently tested in a randomized controlled trial [7]. Following its inclusion in the World Health Organization’s Essential Medicines List (WHO EML) in 2009 and the WHO EML for children in 2013, NECT became the first-line treatment for second stage HAT [8,9]. Since then, the newly developed oral molecule, fexinidazole, received a positive opinion from the European Medicine Agency (EMA) for the treatment of first and second stage HAT in 2018 [10,11]. Nevertheless, NECT remains the first-choice treatment in patients presenting a clinical picture consistent with severe second stage HAT with ≥ 100 white blood cells (WBC)/μL in the cerebrospinal fluid (CSF), in patients who are unable to eat, or in pediatric patients younger than 6 years old or weighing less than 20 kg presenting with > 5 WBC/μL or trypanosomes in the CSF [12–14].

NECT was tested and documented in a phase IIIb clinical trial (NECT-FIELD study) in the DRC from 2009 to 2012 to allow a better understanding of its effectiveness, including a wide population, such as children, pregnant and breastfeeding women. The in-hospital safety profile of NECT and the utilization of concomitant drugs to treat HAT symptoms or comorbidities have already been published by Schmid et al. [15] and Kuemmerle et al. [16]. The purpose of this report is to present the effectiveness results at the end of the 24-month follow-up period.

## Methods

### Ethics statement

This study was conducted in accordance with the ethical principles for medical research involving human subjects, as expressed in the Declaration of Helsinki and its amendment (Version 2008, Seoul, Republic of Korea) available at the time of study conduct. The NECT FIELD study protocol was approved by two ethics committees, Commission d’Ethique de la République Démocratique du Congo (Ministry of Health, Kinshasa, DRC), and the Ethikkommission Beider Basel (formerly EKBB, now EKNZ, Basel, Switzerland) [15].

Eligible patients met the study Investigator or his delegate, who explained the study protocol in detail according to the patient information sheet, and requested written consent from the patient or, in the case of minors, severely ill, or mentally impaired patients unable to fully consent, from her/his parent(s)/guardian(s). Whenever possible (depending on age and level of understanding), the children received the information and their assent was obtained [15].

### Registration

The trial is registered at ClinicalTrials.gov, number NCT00906880 (http://www.clinicaltrials.gov/).

### Study design and participants

We conducted this multicenter, phase IIIb, open-label and single-arm clinical study of nifurtimox eflornithine combination therapy in field conditions in the DRC. Six HAT treatment centers integrated in the health zone reference hospitals of two provinces enrolled patients (former province of Bandundu: Bandundu, Kwamouth and Yasa Bonga centers; former province of East Kasaï: Dipumba, Katanda and Ngandajika centers; to note that we refer herein to the former provinces at the time when the study was conducted). All eligible second stage HAT patients diagnosed at the treatment centers were treated with NECT and followed up every 6 months until 24 months after treatment.

Inclusion criteria, treatment, and outcomes at hospital discharge (treatment safety) are described in detail in the in-hospital safety publication [15]. In brief, all second stage HAT patients admitted to the treatment facilities and routinely diagnosed according to the national guidelines and who gave their Informed Consent for participation, were included in the trial. At inclusion, special attention was given to children and pregnant and breastfeeding women. It was under the Investigator’s decision to include these sub-populations. Exclusion criteria were inability to take oral medication and impossibility to use a nasogastric tube, treatment failure after previous NECT treatment or any other condition for which the Investigator judged that another treatment was warranted. Patients having failed another HAT treatment during the previous two years were also eligible and reported as “previous HAT patients”. Those that may have been treated over two years before their inclusion were considered as new cases and included in the general group. After treatment, all patients discharged alive were eligible for follow-up. The main objective of the follow-up analysis was to assess the effectiveness of NECT at 24 months after treatment and the feasibility of its implementation and rollout under field conditions. The primary endpoint was the proportion of patients discharged alive from the treatment center, which has been described in Schmid et al. 2012 [15]. The secondary endpoint was the clinical cure rate (survival without clinical and/or parasitological signs of HAT) at 24 months after treatment. Safety assessments and adverse event (AE) management were done throughout the follow-up period.

### Clinical outcome definitions

The assessment of treatment outcome at 24 months after NECT administration was based on the criteria recommended by the WHO Informal Consultation [17]. Patient outcomes were termed accordingly: (i) cured—no evidence for trypanosomes in any body fluid examined with a normalized white blood cell count in CSF and/or no evidence of clinical signs and symptoms of HAT; (ii) death—death of patient during treatment or follow-up; death was categorized based on likely or definite cause of death as: HAT, adverse event related to treatment of HAT, adverse event unrelated to HAT and treatment, unknown, and other causes; (iii) relapse—trypanosomes detected in any body fluid or probable relapse—no evidence of trypanosomes but >20 WBC/mm^3^ in the CSF or no evidence of trypanosomes in patients who refuse lumbar puncture and who present with clinical signs of HAT and/or marked deterioration of clinical condition relative to previous evaluations unlikely due to another disease and who, in the opinion of the Investigator, require rescue treatment.

### Follow-up of patients

Follow-up was conducted according to the normal procedures of the DRC HAT control program where patients were asked to return to the treatment center for examinations every 6 months for up to 2 years (6, 12, 18, and 24 months) or whenever the state of health worsened. Reinforced follow-up was organized for all patients at 24 months, and, during the intermediate visits, it was organized for treated children, pregnant or breastfeeding women, and for children born from mothers who were pregnant or breastfeeding during treatment.

At the follow-up examination, patients were tested for the presence of trypanosomes in body fluids (blood and CSF). The CSF was examined for white blood cell count (WBC CSF). The Investigator assessed the state of health of the patients. Reported AEs were documented and treated if necessary. Serious adverse events (SAE) were managed using the best means locally available and were notified to the Health Authorities in DRC and to the Ethics Committees in DRC and Switzerland that reviewed the initial trial proposal. WHO and manufacturers were informed through compiled annual SAE reports. Children who were treated with NECT and children born from mothers who were pregnant or breastfeeding during their NECT treatment were assessed for their growth and development during the entire follow-up period.

Relapses received rescue treatment according to the national policy (i.e., NECT long therapy: 10 days nifurtimox 3 times per day combined with 14 days eflornithine 4 times per day).

### Statistical considerations and analysis

As the trial was open-label and single-arm, the statistical evaluations were descriptive. The results are presented by the sub-populations of interest (center, children below 12 years of age, pregnant or breastfeeding women, and patients with a HAT treatment history). Missing data were not replaced for the analysis.

Time to follow-up intervals were defined at 6, 12, 18, and 24 months according to WHO recommendations [17]. These windows were agreed on to ensure an unbiased data analysis, such that the measured outcomes of a specific patient with a follow-up visit outside the protocol-scheduled time windows could be attributed unequivocally to a specific time window, i.e., 6 months (5–9 months), 12 months (10–16 months), 18 months (17–21 months), and 24 months (≥ 22 months).

The main study population for the effectiveness analysis was the modified intention to treat (mITT) population. It consisted of all patients who received at least one dose of study drug and attended at least one follow-up visit (or reached a fatality endpoint during the hospitalization phase). The patients were classified according to the clinical response criterion available at the last visit.

The secondary study population was the per protocol to follow-up (PP2) population, which included all patients who arrived at a study endpoint (death or relapse) or completed follow-up at 24 months.

The effectiveness endpoint was the clinical cure rate at 24 months after treatment (cured versus not cured, i.e., probable relapse, relapse, or death) at the 24 months follow-up evaluation.

Sensitivity analysis was performed on the intention to treat (ITT) population by applying two extreme hypotheses to classify completely lost to follow-up (CLTFU) patients, i.e., by considering all CLTFU as cured and as failures.

The analyses were carried out using SAS software versions 9.1 & 9.2 and STATA 12.

## Results

We report here the follow-up results following hospitalization and treatment with NECT. Screening, baseline, treatment, and in-hospital safety and efficacy results were published in Schmid et al [15].

**Fig 1:**
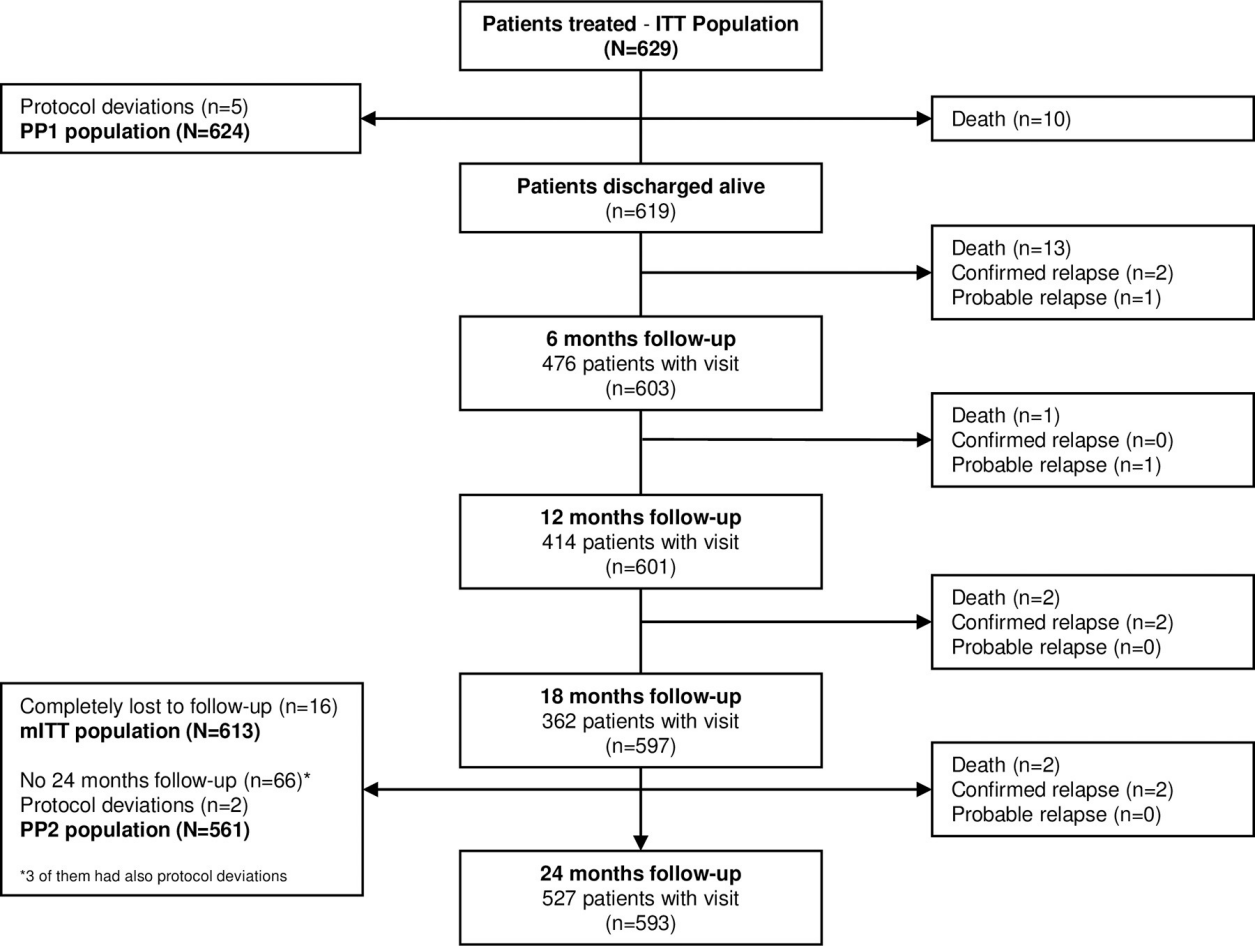
Flow diagram of the study population Screening flowchart is shown in the in-hospital publication [15].

Overall, 629 second stage HAT patients were enrolled and treated between May 2009 and May 2010. After treatment, 619 patients who were discharged alive were followed up for 24 months. The last follow-up visit occurred in September 2012 (Fig 1). The ITT and PP1 populations were used for the in-hospital and efficacy result calculations [15]. For the effectiveness evaluation, 16 patients were excluded from the mITT population due to no follow-up data (completely lost to follow-up, CLTFU). A total of 68 patients were excluded from the PP2 population; 66 patients had no 24-month follow-up visit (3 of them had also protocol deviations) and 2 patients had a protocol deviation (Fig 1).

Table 1 shows a summary of the baseline characteristics, the discharge results from the hospitalization period, and the follow-up compliance. During follow-up, 16 out of 629 (2.5%) patients were completely lost. The reasons remain unknown, it is likely that these patients moved away from the study provinces. The participation varied between follow-up visits and between subpopulations of interest. The highest follow-up participation was seen at the 6- and 24-month follow-up examinations. Of note is the fact that the last visit included active follow-up with a specific budget, as the previous visits aimed to reflect “real life” conditions and there was no dedicated extra resources nor effort made to trace missing patients until the 24-month visit period. The follow-up examinations were mainly carried out at the treatment center. However, for various reasons, some patients were seen in their village, and for a few patients only oral reports by family members or neighbors were available (8 (1.3%) at 24 months or less, depending on the follow-up visit). Follow-up compliance also varied between the subpopulations analyzed, mainly between the different study sites, with a higher follow-up coverage in the Bandundu province (for follow-up coverages, see Tables A and B in S1 Text).

**Table 1:** Patient characteristics on admission, and during treatment and follow-up.

Number of patients n (%)^1^	All patients (N = 629)
Demographic characteristics on admission	
Age (years), median (range)	28 (0.5–77)
Children (0–11 years)	100 (15.9)
Adolescents / Adults (>11years)	529 (84.1)
Female	275 (43.7)
Breastfeeding women	33 (5.2)
Pregnant women	14 (2.2)
Previous HAT patients^2^	84 (13.4)
Drugs received:	
Eflornithine	43 (6.8)
Melarsoprol	18 (2.9)
Combinations	4 (0.6)
Pentamidine	19 (3.0)
Body-mass index^3^ <18·5 kg/m^2^	172 (41.9)
Parasitology findings on admission	
Presence of trypanosomes	558 (88.7)
In lymph nodes	218 (34.7)
In blood	162 (25.8)
In CSF	341 (54.2)
Leucocyte count in CSF, median (range)	153 (2–3040)
6–20 leucocytes/μL	94 (14.9)
21–99 leucocytes/μL	137 (21.8)
>100 leucocytes/μL	397 (63.1)
Clinical characteristics on admission	
Karnofski index^4^, median (range)	70 (20–90)
Altered or bad general state of health	502 (79.8)
HAT Symptoms	
Sleeping disorders (insomnia, somnolence)	503 (80.0)
Headache	472 (75.0)
Behavioral disorder (aggressive, anxious, depressive)	208 (33.1)
Hospitalization period	
Number of patients treated	629 (100)
Treatment adherence^5^	602 (95.7)
Number of deaths	10 (1.6)
Number of patients discharged alive	619 (98.4)
Follow-up period	
Number of patients to follow up	619 (98.4)
Completely lost to FU (missing all 4 visits)	16 (2.5)
Patients with partial FU (1–3 visits)	327 (52.0)
Patients with complete FU (4 visits)	265 (42.1)
Patients with 24 months FU visit completed	527 (83.8)

Abbreviations: CSF, cerebrospinal fluid; FU, follow-up; HAT, human African trypanosomiasis.

^1^unless otherwise indicated

^2^patients having failed another HAT treatment within the previous 2 years.

^3^BMI was calculated only for patients >12 years of age (118 patients with BMI unknown; N = 411).

^4^The Karnofsky index runs from 100 to 0, where 100 is normal health and 0 is death.

^5^treatment adherence of patients having received the complete treatment as per protocol (i.e., 30 doses nifurtimox & 14 doses eflornithine).

Over the follow-up period, the general state of health improved for nearly all examined patients at each visit (97% at 6 months follow-up to 99% at the 24-month follow-up visit). The evolution of the general state of health after treatment was comparable for all described sub-populations except for patients at the Ngandajika center who seemed to recover slightly slower.

White blood cells in the CSF normalized during the follow-up period. At 6 months, none of the patients had more than 100 cells in the CSF, with the majority (82%) displaying between 0 and 5 cells. At 12, 18, and 24 months, nearly all the patients’ white blood cell counts in the CSF were normalized between 0 and 5 white blood cells (95%, 98%, and 99% of the examined patients, respectively). In addition, five patients presented trypanosomes in the CSF during follow-up; two at the 6-month, one at the 18-month, and two at the 24-month follow-up visits (for follow-up diagnosis test details, see Tables C and D in S1 Text; for patient follow-up evolution details see Tables E and F in S1 Text).

In total, 36 patients reached an endpoint before the 24-month follow-up visit. Ten patients died during treatment, eighteen during the follow-up period, and eight relapsed after hospital discharge. Of those latter, six were considered as true relapses with trypanosomes found in the CSF, and two as probable relapses based on elevated white blood cell counts in the CSF. These patients received a rescue treatment of 14 days eflornithine and 10 days nifurtimox. This rescue treatment is known as “NECT long” and is proposed in the national treatment guidelines for areas with known melarsoprol resistance. As presented in Table 2, the overall cure rate of the mITT population was 94.1% (95% CI 91.8 to 95.7%) at 24 months, with similar results among the subpopulations of interest (age groups, region, and with previous HAT history). These main effectiveness results in the mITT population were confirmed by the PP2 population analysis, by the sensitivity analysis where 16 completely lost to follow-up patients (who were excluded from the mITT population) were either considered as cured or as failures.

**Table 2:** Efficacy and effectiveness indicators.

Efficacy and effectiveness indicators	n	N	%	(95% CI)
Hospitalization period				
Number of patients (ITT population)	629	629	100	
In-hospital fatalities	10	629	1.6	
Discharged alive (primary endpoint)	619	629	98.4	(97.1–99.1)
Follow-up period				
Fatalities	18	629	2.8	
Relapses (confirmed and probable relapses^1^)	8	629	1.3	
At 24 months				
Clinical cure at 24 months (effectiveness, secondary endpoint)				
mITT population	577	613	94.1	(91.8–95.7)
PP2 population	525	561	93.6	(91.0–95.3)
Sensitivity analysis				
ITT CLTFU considered as cured	593	629	94.3	(92.0–95.8)
ITT CLTFU considered as failures	577	629	91.7	(89.3–93.8)
Probability of cure per subpopulation of interest				
Overall, mITT population	577	613	93.9	(91.6–95.6)
Children 0–11 years	93	99	93.1	(85.0–96.9)
Pregnant or breastfeeding women	46	47	97.9	(85.8–99.7)
Other patients	438	467	93.6	(91.0–95.5)
Previous HAT history				
Within 2 years^2^	77	82	93.3	(84.5–97.2)
Probability of cure per site				
Kasai Oriental Province				
Dipumba	129	138	93.2	(87.2–96.4)
Katanda	121	132	91.4	(85.0–95.2)
Ngandajika	84	89	94.2	(86.5–97.5)
Bandundu Province				
Bandundu	94	98	95.9	(89.4–98.4)
Kwamouth	94	97	96.9	(90.7–99.0)
Yasa Bonga	55	59	93.2	(82.9–97.4)

Abbreviations: ITT, intention to treat; mITT, modified intention to treat; CLTFU, completely lost to follow up.

^1^confirmed relapses (patients with detected trypanosomes in any body fluid), probable relapses (patients with no evidence of trypanosomes but >20 WBC/mm3 in CSF or clinical evidence).

^2^Previous HAT history within 2 years, can be considered as previous treatment failure.

All the safety results of the hospitalization period are described in Schmid et al. [15]. During the follow-up period, the general state of health improved for nearly all patients (97% at 6 months follow-up to 99% at the 24-month follow-up visit). The overall safety results are presented in Table 3. The percentage of patients with at least one reported AE during the follow-up period was 31.3%. Details of the AEs during the follow-up are given in Table 4. Reporting rates in the study sites were very heterogeneous, ranging from 3.4% of patients with at least one AE in Yasa Bonga to 62.3% in Dipumba. Details concerning administration of concomitant medications to treat AEs, HAT symptoms, and comorbidities are reported elsewhere [16].

**Table 3:** Safety characteristics.

Safety characteristics Number of patients n (%)	All patients	Children below 12 years	Pregnant or breastfeeding women	Adolescents/adults^3^
Overall safety (treatment and follow-up period)				
ITT population	629	100	47	482
Patient with any adverse event^1^	597 (94.9)	98 (98.0)	44 (93.6)	455 (94.4)
Death	28 (4.5)	3 (3.0)	1 (2.1)	24 (5.0)
Death related^2^ to treatment	11 (1.7)	0	1 (2.1)	10 (2.1)
Non-fatal SAEs	33 (5.2)	5 (5.0)	3 (6.4)	26 (5.4)
Non-fatal SAEs related^2^ to treatment	22 (3.5)	2 (2.0)	3 (6.4)	17 (3.5)
Safety overview treatment period				
ITT population	629	100	47	482
Patient with any adverse event^1^	578 (91.9)	92 (92.0)	42 (89.4)	444 (92.1)
Death	10 (1.6)	0	1 (2.1)	9 (1.9)
Death related^2^ to treatment	9 (1.4)	0	1 (2.1)	8 (1.7)
Non-fatal SAEs	22 (3.5)	2 (2.0)	3 (6.4)	17 (3.5)
Non-fatal SAEs related^2^ to treatment	17 (2.3)	1 (1.0)	3 (6.4)	13 (2.7)
Safety overview during the follow-up period				
mITT population	613	99	47	467
Patient with any adverse event^1^	192 (31.2)	29 (29.3)	18 (38.3)	145 (31.0)
Death	18 (2.9)	3 (3.0)	0	15 (3.2)
Death related^2^ to treatment	2 (0.3)	0	0	2 (0.4)
Non-fatal SAEs	12 (2.0)	3 (3.0)	0	10 (2.1)
Non-fatal SAEs related^2^ to treatment	5 (0.8)	1 (1.0)	0	4 (0.9)

Abbreviations: ITT, intention to treat; mITT, modified intention to treat; SAE, serious adverse event

^1^Except fatalities and SAEs

^2^Assessed as possibly or probably related to NECT (causality synthesis)

^3^Except pregnant or breastfeeding women

**Table 4:** Most frequent and relevant adverse events during follow-up.

Safety characteristics Number of patients n (%)	All patients	Children below 12 years	Pregnant or breastfeeding women	Adolescents/adults^1^
Adverse events^2^				
mITT population	613	99	47	467
Nervous system disorders	59 (9.6)	11 (11.1)	5 (10.6)	43 (9.2)
Headache	46 (7.5)	6 (6.1)	5 (10.6)	35 (7.5)
Convulsions	6 (1.0)	5 (5.1)	0	1 (0.2)
Infections and infestations	52 (8.5)	8 (8.1)	6 (12.8)	38 (8.1)
Malaria	19 (3.1)	3 (3.0)	5 (10.6)	11 (2.4)
Influenza	10 (1.6)	0	1 (2.1)	9 (1.9)
Cold	8 (1.3)	2 (2.0)	0	6 (1.3)
Gastrointestinal disorders	37 (6.0)	6 (6.1)	5 (10.6)	26 (5.6)
Diarrhea	11 (1.8)	3 (3.0)	2 (4.3)	6 (1.3)
General disorders and administration site conditions	37 (6.0)	8 (8.1)	0	29 (6.2)
Fever	29 (4.7)	8 (8.1)	0	21 (4.5)
Musculoskeletal and connective tissue disorders	34 (5.5)	3 (3.0)	0	31 (6.6)
Lumbago	10 (1.6)	0	0	10 (2.1)
Pain in the inferior extremities	6 (1.0)	0	0	6 (1.3)
Psychiatric disorders	24 (3.9)	6 (6.1)	0	18 (3.9)
Insomnia	10 (1.6)	1 (1.0)	0	9 (1.9)
Respiratory, thoracic, and mediastinal disorders	16 (2.6)	1 (1.0)	0	15 (3.2)
Dry cough	4 (0.7)	0	0	4 (0.9)
Productive cough	4 (0.7)	0	0	4 (0.9)
Cough	3 (0.5)	1 (1.0)	0	0
Skin and sub-cutaneous tissue disorders	21 (3.4)	2 (2.0)	0	19 (4.1)
Pruritus	16 (2.6)	2 (2.0)	0	14 (3.0)

Abbreviations: mITT, modified intention to treat

^1^Except pregnant or breastfeeding women

^2^Coded according to the MedDRA dictionary into System Organ Class (SOC) and Lower Level Term (LLT)

During follow-up, 12 patients had a non-fatal SAE, 6 of which were considered as life-threatening (see Table 3; all non-fatal SAEs that occurred during the treatment and follow-up period are shown in Tables G and H in S1 Text). The causes of non-fatal SAE were diverse, such as infections (3 patients), gastrointestinal disorders (3 patients), anemia (1 patient), convulsions (1 patient), hypotension (1 patient), surgery (ovarian cyst and appendicectomy in 2 patients), and poly-traumatic injuries (1 patient). Of these non-fatal SAEs, 5 were considered to be possibly related to the study drug (3 of them were infections, 1 anemia, and 1 upper gastrointestinal hemorrhage). The others were all assessed as not related (unlikely or not related).

During follow-up, 18 patients died of various causes such as septic shock (2 patients), cerebral malaria (1 patient), pneumopathy (1 patient), hypovolemic shock (1 patient), metabolic disorders (2 patients), gastrointestinal disorders (2 patients), injuries (1 patient), and unspecified death (8 patients) (see Table 3; all death cases that occurred during the treatment and follow-up period are shown in Tables I and J in S1 Text). Of these, 16 deaths were assessed as unlikely or not related to the study treatment while two were assessed as probably related and possibly related.

As reported in the previous in-hospital safety and efficacy publication by Schmid et al [15], 13 pregnant women were discharged alive and were eligible for follow-up (one pregnant woman died during the in-hospitalization period). All 13 women gave birth to a live baby. No anomaly was reported. The general condition, as well the physical and motor development and the language development of the children of women treated with NECT during the pregnancy, but also during breastfeeding were good and evolved adequately over two years.

## Discussion

Following the publication of the randomized phase III multicentric clinical trial evaluating NECT versus eflornithine that reported non-inferior efficacy and an appropriate safety profile [7], NECT was rapidly endorsed by WHO and endemic countries, becoming the first line treatment for second stage HAT from 2009 [8–9]. Nevertheless, data on NECT’s clinical tolerability, effectiveness, and feasibility in field conditions and in vulnerable populations such as children, pregnant or breastfeeding women, and patients who had been previously treated for HAT (i.e., previous treated HAT infection within 2 years before enrolment into this study) due to relapses, were still lacking. In response to this knowledge gap, the NECT-FIELD study was conducted in six HAT treatment centers in the DRC that varied in their settings, capabilities, and experience; i.e., urban and rural, accessible and remote, clinical trial experienced and unexperienced centers.

The proportion of patients considered as cured 24 months after treatment with NECT (main effectiveness endpoint, mITT population) was 94.1% and was in line with other publications relating results of controlled clinical trials [7,11,18] and other reports relating early development results [19,20], or monitoring field use [21,22]. Failure was defined as all confirmed deaths, independent of their cause or relationship to the product, and all relapses, confirmed with microscopic detection of trypanosomes, or probable, due to the increase of white blood cells detected in the cerebrospinal fluid. It was decided to examine those completely lost to follow-up patients in a sensitivity analysis, considering both extreme possibilities, either all of them as failure or all as success. The overall results did not vary markedly between the various analyzed populations (mITT, PP2) and sensitivity analysis (ITT CLTFU failure and ITT CLTFU cure), all had a cure rate of between 91.7% and 94.3%. Neither did the rates markedly vary between the defined subpopulations, i.e., children, pregnant and breastfeeding women, and patients who had been previously treated for HAT. In our study, no matters of concern arose with similar dosages of the treatment applied to children as for adults. While higher doses of eflornithine monotherapy have been recommended for children by Van Nieuwenhove [23] and by Milord et al [24], Priotto observed that effectiveness did not increase with higher doses [25].

During the follow-up period, SAEs, including re-hospitalization (12/613) and fatalities (18/613), were systematically reported. The AEs were not systematically collected unless spontaneously reported by the patient or directly assessed by the Investigator at the follow-up visit. The overall fatality rate was 4.5%, 2.9% for the follow-up period and 1.6% for the in-hospital period as previously reported by Schmid et al. [15]. Case fatality during the follow-up period was consistent with previously published reports [7,11,18–22]. Five SAEs (death and non-fatal) of infectious origin and one of anemia were reported. They all occurred within three months of the end of the treatment. The bone marrow suppressive effect of eflornithine may have played a role in these few cases of infections and anemia, although such afflictions are common in this type of setting.

The long-term AE profile during the 24-month follow-up period consisted mainly in headaches, fever, malaria, and diarrhea and seemed to be in line with the general state of health of a rural central African population. No safety and development concerns arose for children of women treated during pregnancy or during breastfeeding.

Our study has some limitations. First, the number of lost to follow-up (completely lost, without any visit or partly lost) was slightly higher than in the previously conducted controlled trials [7]. Nevertheless, mitigation strategies and highly motivated study teams resulted in follow-up compliance that was still high compared to the observational setting. The sensitivity analysis of those completely lost to follow-up as either cured or treatment failures provides reassurance in the reported effectiveness results.

Secondly, effectiveness and safety results in the vulnerable sub-populations must be considered with caution, as the sample sizes of children, pregnant and breastfeeding women, and patients who had previously received HAT treatment were small and, consequently, the ability to calculate effectiveness and detect uncommon adverse events in these subgroups was limited.

Third, during follow-up, the AEs were not systematically collected unless spontaneously reported by the patient or directly assessed by the Investigator at the follow-up visit examination, while the SAEs, including all death cases, were systematically reported, which might have resulted in an under-reporting of non-serious AEs. AE reporting rates during follow-up were very heterogeneous. This is most probably due to different reporting procedures in the study sites during follow-up, as during the hospitalization phase AE were similarly reported among centers. Due to this heterogeneity of reporting during follow-up, AEs in certain centers might be under- or misreported, possibly creating a biased picture of AE occurrence, with underestimated frequencies. It might therefore be worth implementing more stringent procedures to report AEs during follow-up to mitigate the under-reporting of AEs.

NECT seems to have no impact on the physical development of children, from women treated during the pregnancy, and breastfeeding mothers. Nevertheless, these results must be interpreted cautiously due to the small sample size.

In conclusion, NECT has been shown to be effective and safe for use in remote and rural sleeping sickness facilities in endemic countries. Use of NECT under field conditions was found to be feasible, provided that staff are thoroughly trained, especially in the application and hygiene of intravenous treatment, and that logistical hurdles are overcome in locations where road, boat, or plane access are difficult. In the current context of HAT elimination [26], NECT plays an important role alongside the newly approved fexinidazole treatment [10,12] as it remains the first line treatment in patients with severe second stage HAT, defined as presenting over 100 WBC/ μL CSF, in patients who are unable to eat, or in pediatric patients younger than 6 years old or weighting less than 20 kg [13,14]. In our study, all 74 children below 20 kg weight, including all those below 6 years, showed a similar effectiveness of 94.6% (74/99) (95% CI 86.7–98.5). Among the 385 patients with more than 100 WBC/ μL CSF, the measured effectiveness was of 93.0% (358/385) (95% CI 90.0–95.33), and 89.5% (34/38) (95% CI 75.2–97.1) for the children subgroup with more than 100 WBC/ μL CSF. Nevertheless, specific vulnerable sub-populations, including children, pregnant and breastfeeding women, and patients with specific co-morbidities, need to be included in further studies to collect evidence in the context of HAT elimination.

## Supporting information

S1 Text Table A. Disposition of patient follow up by subpopulation of interest. Table B. Disposition of patient follow up by Centre.Table C. Specific diagnostic tests for HAT during the follow up and the evolution of the white blood cell (WBC) counts in CSF by sub-population of interest. Table D. Specific diagnostic tests for HAT during the follow up and the evolution of the white blood cell (WBC) counts in CSF by centre. Table E. Summary of the patient evolution throughout the follow up period by sub-population of interest. Table F. Summary of the patient evolution throughout the follow up period by centre. Table G. Patients with non-fatal serious adverse events during the treatment period. Table H. Patients with non-fatal serious adverse events during the follow-up period. Table I. Patients who died during the treatment period. Table J. Patients who died during the follow-up period.
(DOCX)
