# Supplementary material for: Effectiveness of Nifurtimox Eflornithine Combination Therapy (NECT) in T. b. gambiense second stage sleeping sickness patients in the Democratic Republic of Congo: Report from a field study
Source: PLoS Negl Trop Dis. 2021 Nov 8;15(11):e0009903. doi: 10.1371/journal.pntd.0009903 (PMC8601604; doi:10.1371/journal.pntd.0009903)
Supplement: S1 Text — Table A. Disposition of patient follow up by subpopulation of interest. Table B. Disposition of patient follow up by Centre.Table C. Specific diagnostic tests for HAT during the follow up and the evolution of the white blood cell (WBC) counts in CSF by sub-population of interest. Table D. Specific diagnostic tests for HAT during the follow up and the evolution of the white blood cell (WBC) counts in CSF by centre. Table E. Summary of the patient evolution throughout the follow up period by sub-population of interest. Table F. Summary of the patient evolution throughout the follow up period by centre. Table G. Patients with non-fatal serious adverse events during the treatment period. Table H. Patients with non-fatal serious adverse events during the follow-up period. Table I. Patients who died during the treatment period. Table J. Patients who died during the follow-up period. (DOCX) [file pntd.0009903.s001.docx]

Supporting information

**S1 Text**

Contents

[Table A. Disposition of patient follow up by subpopulation of interest 2](#_Toc85112672)

[Table B. Disposition of patient follow up by Centre 3](#_Toc85112673)

[Table C. Specific diagnostic tests for HAT during the follow up and the evolution of the white blood cell (WBC) counts in CSF by sub-population of interest 4](#_Toc85112674)

[Table D. Specific diagnostic tests for HAT during the follow up and the evolution of the white blood cell (WBC) counts in CSF by centre 5](#_Toc85112675)

[Table E. Summary of the patient evolution throughout the follow up period by sub-population of interest 6](#_Toc85112676)

[Table F. Summary of the patient evolution throughout the follow up period by centre 7](#_Toc85112677)

[Table G. Patients with non-fatal serious adverse events during the treatment period 8](#_Toc85112678)

[Table H. Patients with non-fatal serious adverse events during the follow-up period 9](#_Toc85112679)

[Table I. Patients who died during the treatment period 10](#_Toc85112680)

[Table J. Patients who died during the follow-up period 11](#_Toc85112681)

# Table A. Disposition of patient follow up by subpopulation of interest

# Table B. Disposition of patient follow up by Centre

# Table C. Specific diagnostic tests for HAT during the follow up and the evolution of the white blood cell (WBC) counts in CSF by sub-population of interest

# Table D. Specific diagnostic tests for HAT during the follow up and the evolution of the white blood cell (WBC) counts in CSF by centre

# Table E. Summary of the patient evolution throughout the follow up period by sub-population of interest

# Table F. Summary of the patient evolution throughout the follow up period by centre

# Table G. Patients with non-fatal serious adverse events during the treatment period

# Table H. Patients with non-fatal serious adverse events during the follow-up period

# Table I. Patients who died during the treatment period

# Table J. Patients who died during the follow-up period
